# Chronic disease mortality in rural and urban residents in Hubei Province, China, 2008–2010

**DOI:** 10.1186/1471-2458-13-713

**Published:** 2013-08-02

**Authors:** Liwei Cheng, Li Tan, Lan Zhang, Sheng Wei, Li Liu, Lu Long, Jie Zhang, Yaqiong Wu, Qingjun Zhang, Shaofa Nie

**Affiliations:** 1 Department of Epidemiology and Biostatistics, School of Public Health, Tongji Medical College of Huazhong University of Science and Technology, 13 Hangkong Road, Qiaokou District, Wuhan, Hubei Province 430030, PR China; 2 Hubei Province Centers for Disease Control and Prevention (CDC), 6 Zhuo-dao-quan-bei Road, Hongshan District, Wuhan, Hubei Province 430079, PR China

## Abstract

**Background:**

Chronic non-communicable diseases have become the major cause of death in China. This study describes and compares chronic disease mortality between urban and rural residents in Hubei Province, central China.

**Methods:**

Death records of all individuals aged 15 years and over who died from 2008 through 2010 in Hubei were obtained from the Disease Surveillance Points system maintained by the Hubei Province Centers for Disease Control and Prevention. Average annual mortality, standardized death rates, years of potential life lost (YLL), average years of potential life lost (AYLL) and rates of life lost were calculated for urban and rural residents. Standardized rate ratios (SRR) were calculated to compare the death rates between urban and rural areas.

**Results:**

A total of 86.2% of deaths were attributed to chronic non-communicable diseases in Hubei. Cerebrovascular diseases, ischemic heart disease and neoplasms were the main leading causes in both urban and rural areas, and the mortality rates were higher among rural residents. Lung cancer was the principal cause of mortality from cancer among urban and rural residents, and stomach cancer and liver cancer were more common in rural than urban areas. Breast cancer mortality among women in rural areas was lower than in urban areas (SRR=0.73, 95% CI=0.63–0.85). The standardized mortality for chronic lower respiratory disease among men in rural areas was higher than in urban areas (SRR=4.05, 95% CI=3.82–4.29). Among men, total AYLL from liver cancer and other diseases of liver were remarkably higher than other causes in urban and rural areas. Among women the highest AYLL were due to breast cancer in both urban and rural areas.

**Conclusions:**

Chronic diseases were the major cause of death in Hubei Province. While circulatory system diseases were the leading causes in both urban and rural areas, our study highlights that attention should also be paid to breast cancer among women and chronic lower respiratory disease among rural residents. It is important that governments focus on this public health issue and develop preventive strategies to reduce morbidity and premature mortality from chronic non-communicable diseases.

## Background

Chronic non-communicable diseases (NCDs) are a global health problem and a threat to human health and development. Among both men and women, the majority of deaths worldwide are due to NCDs, which account for about six of ten deaths globally [1]. In recent decades, the healthcare system in China has significantly improved, and the health status of China’s population has changed as a consequence of economic and social development [2,3]. From 1990 to 2005, the life expectancy of men and women in China increased through health interventions such as increased vaccination coverage, improved access to medical care, and improved social and living standards [4]. Accompanying the rapid growth of the national economy and changes in environment and lifestyle, the predominant cause of mortality in China has shifted from infectious diseases to NCDs [5,6]. National data show that NCDs have become increasingly common causes of death in recent decades [7]. In 2008, data from the Fourth Chinese National Health Services Survey showed that about 82% of deaths were caused by NCDs [8], and it is predicted that deaths attributable to NCDs will rise to 85% by 2020 [9].

The WHO 2008–2013 action plan for NCDs focuses on four NCDs: cardiovascular diseases, diabetes, cancer and chronic respiratory disease [10]. These four NCDs share risk factors such as tobacco use, physical inactivity, unhealthy diets and harmful use of alcohol [11]. Health behaviors are affected by socioeconomic status, such as income and education, sociodemographic factors, and cultural values [12]. There are significant differences in socioeconomic and health status among different regions in China. Despite rapid economic growth and a continued trend toward urbanization, the gap between urban and rural income has increased over the past few decades. In 2010, rural residents had an annual average per capita disposable income of US $898, less than a third of the average per capital disposable income of urban residents (US $2,900). Studies also indicate that patterns in causes of death vary between urban and rural areas, and the gap in health status between urban and rural residents is widening [5,13]. However, comparative data on chronic diseases mortality between urban and rural residents in China is sparse.

Measures of mortality and cause of death are important for public health programs and policies [14]. In this article, we described and compared chronic disease mortality between urban and rural residents in Hubei Province from 2008 to 2010, using data from the Disease Surveillance Points (DSPs) system of Hubei Province. Our study is an essential part of public health services and provides information concerning the main health problems to policy makers and program managers in making informed policy decisions to improve the effectiveness of health investments.

## Methods

### Data source

In China, complete registration and medical certification of deaths is logistically and financially unattainable at present. However, mortality registration systems such as the Ministry of Health-Vital Registration system (MOH-VR system) and Chinese CDC-Disease Surveillance Points (DSPs) system have been established. The coverage of the MOH-VR system is biased toward the urban and better-off populations of eastern China, which does not represent the national population. In contrast, the DSPs system uses random sampling to establish a nationally representative sample [15]. In this study, we used data collected through the DSPs system maintained by the Hubei Province Centers for Disease Control and Prevention. These data are not publicly available, but we have the permission from the Hubei Province CDC to publish the results.

Hubei Province is an intermediately economically developed province located in central China (29°05’-33°20’N, 108°21’-116°07’E), with a population density of 308/km^2^ in 2010 [16]. The gross regional product of Hubei Province was US $235.9 billion in 2010, and the primary industry, secondary industry and tertiary industry accounted for 13.5%, 48.6% and 37.9%. With a total population of 57.2 million, the per capita gross domestic product (GDP) of the province was US $4,123 in 2010 and US $5,285 in 2011. The difference in average per capita disposable income between rural and urban areas was about fourfold. Data of the Fourth Chinese National Health Services Survey showed that the chronic disease prevalence was 193.3 per 1000 individuals in urban areas, and 118.0 per 1000 individuals in rural areas in the province [17].

A detailed description of the history and organization of the DSPs system in China has been previously described by Yang et al. [15]. About 50% deaths in urban areas and 80% deaths in rural areas occurred at home. For deaths occurring in hospitals, a death certificate was filled in by the physician who attended the death. For deaths occurring at home, a village health worker or community clinician reports the event to the Prevention Unit at the township hospital or Community Health Center. A staff member from the unit visits the household and completes a death certificate based on a description of symptoms from family members and available documents from recent contacts with health services. The DSPs staff members at a local Center for Disease Control and Prevention office collect the death certificates and enter the information into the DSPs system. Every three years, under-reporting is estimated by a survey that covers 5% of the surveillance population, and mortality estimates are adjusted accordingly.

A total of four districts and six counties were selected for death registration in 2008. Based on the multistage stratified cluster sampling method considering socioeconomic conditions and population size, this included 10% of the total population in Hubei. In 2009, an urban district was added into the system as a new surveillance site. The urban data from the DSPs system were mainly from a non-agricultural population of large and middle size cities, and the rural data were mainly from an agriculture population, as described by Liu et al. [18].

### Data analysis

Records included all people aged 15 years and over who died from 2008 through 2010 in the surveillance sites in Hubei. Causes of death were classified according to the International Classification of Diseases, 10th Revision. The chronic diseases included were cerebrovascular diseases, ischemic heart disease, neoplasms, chronic lower respiratory diseases, diabetes mellitus and diseases of the liver.

Average annual cause-specific death rates were calculated by the total number of deaths from a specified cause per 100,000 person-years, for different sex and age groups. The standardized death rates were calculated by the direct standardization method. The reference population for both men and women was the national population in 2010 obtained from the Sixth National Population Census. Standardized rate ratios (SRR) and 95% confidence intervals to compare death rates between urban and rural areas were calculated, with urban as the control group. Years of potential life lost (YLL), average years of potential life lost (AYLL) and rates of life lost were calculated to estimate the burden of chronic diseases in the province. The average life expectancy in China was 74.8 years at the end of 2010; in Hubei Province it was 74.9 years [19]. Thus, we used 75 years as life expectancy to calculate the indicators.

Rates and ratios were calculated as follows. YLL = ∑ a_*i*_*d*_*i*_, *a*_*i*_ = L − (*x*_*i*_ + 0.5), where *a*_*i*_ is the number of years of life lost by an individual who dies in the *i*th age group, *L* is the cxxlife expectancy, *x*_*i*_ is the average age of the *i*th age group, and *d*_*i*_ is the number of observed deaths in the *i*th age group. AYLL = *YLL*/∑*d*_*i*_, where ∑ *d*_*i*_ is the total number of observed deaths by the specific cause. Rates of life lost per 1,000 individuals were calculated by (*YLL*/*n*) × 1, 000, where *n* is the number of people in the population under 75 years.

Microsoft Excel 2007/2003 and PASW statistics 18.0 (SPSS, Chicago, IL, USA) were used for data management and statistical analysis.

## Results

Sex, age distribution, crude mortality and standardized mortality in urban and rural areas in each year in Hubei Province are shown in Table 1. The proportion of people aged over 65 years in urban areas was higher than in rural areas, and the standardized mortality was higher among rural residents. The average annual mortality was 5.90 per 1000 individuals, and the standardized mortality rate was 5.32 per 1000 individuals. NCDs accounted for 86.2% of total deaths in citizens over 15 years of age. Mortality for males was higher than females in all age groups, and older age led to an increase in mortality for both men and women (Table 2).

**Table 1:** Sex, age distribution and mortality in urban and rural areas of Hubei, 2008–2010

	**Year**	**Total**	**Male (%)**	**Female (%)**	**15–64 years**	**≥65 years**	**Crude death**	**Standardized death**
		**population**			**(%)**	**(%)**	rate*	rate*
**Urban**	2008	1,362,596	50.93	49.07	75.02	12.01	6.33	5.19
	2009	1,902,613	50.58	49.42	76.10	12.06	6.48	5.26
	2010	1,925,498	50.31	49.69	77.17	12.05	6.35	5.13
**Rural**	2008	4,696,951	52.16	47.84	70.82	7.13	5.69	6.71
	2009	4,759,722	52.19	47.81	74.70	7.37	5.74	6.53
	2010	4,716,511	52.07	47.93	74.54	7.44	5.79	6.55
**Total**		19,363,891	51.72	48.28	74.13	8.58	5.90	5.32

* per 1,000 individuals.

**Table 2:** Cause-specific mortality rate (per 100,000) by sex and age group in Hubei, 2008–2010

**Diseases**	**Male**			**Female**		
	**15–39 years**	**40–64 years**	**≥65 years**	**15–39 years**	**40–64 years**	**≥65 years**
**Circulatory system (I00-I99)**	10.11	245.58	3126.49	4.41	140.97	2668.55
Cerebrovascular disease	4.27	149.72	1761.30	1.74	87.86	1431.58
Ischemic heart disease	3.44	56.74	669.53	1.21	26.91	596.92
**Neoplasms (C00-D48)**	17.36	255.02	1307.74	11.23	130.30	655.68
Lung	1.66	65.83	412.20	0.70	20.03	143.54
Liver	7.84	78.09	220.19	1.86	22.18	104.86
Stomach	1.06	39.57	222.64	1.04	16.59	113.21
Colon and rectum	0.72	12.50	78.84	0.82	8.28	57.82
Esophagus	0.16	16.56	108.07	0.12	4.05	42.32
Breast	0.00	0.38	0.74	1.28	14.14	28.71
**Respiratory system (J00-J99)**	0.76	24.58	918.57	0.77	12.03	681.02
Chronic lower respiratory disease	0.22	20.81	800.34	0.29	9.84	577.78
**Digestive system (K00-K93)**	1.64	21.32	118.70	0.53	7.60	83.43
Diseases of liver	1.26	15.66	53.15	0.34	5.01	34.91
**Endocrine, nutritional, and metabolic diseases (E00-E90)**	0.70	9.05	82.68	0.51	9.06	100.14
Diabetes mellitus	0.49	8.60	78.99	0.39	8.55	95.42

The number of deaths and mortality rates of each chronic disease in urban and rural areas are shown in Tables 3 and 4. Cerebrovascular diseases, ischemic heart disease and neoplasms were the main leading causes in both urban and rural areas. For neoplasms, lung cancer was the principal cause of mortality among both urban and rural residents. Stomach cancer and liver cancer were more common in rural than in urban areas. In men, age standardized death rates due to most chronic diseases were higher in rural areas than in urban areas (Table 3). The death rates from cerebrovascular disease (SRR=2.24, 95% CI=2.16–2.31), stomach cancer (SRR=2.47, 95% CI=2.25–2.70), and chronic lower respiratory disease (SRR=4.05, 95% CI=3.82–4.29) among men in rural areas was remarkably higher than those in urban areas. Conversely, death rates from lung cancer, colon and rectal cancer and diabetes mellitus were relatively higher in urban areas among both men and women. Among women, breast cancer mortality in rural areas was lower than in urban areas (SRR=0.73, 95% CI=0.63–0.85) (Table 4).

**Table 3:** Mortality rate of chronic disease among men in urban and rural areas in Hubei, 2008–2010

**Diseases**	**Urban**		**Rural**		**SRR (95% CI)**
	**Crude death rate**	**Standardized death rate**	**Crude death rate**	**Standardized death rate**	
	**(per 100,000)**	**(per 100,000)**	**(per 100,000)**	**(per 100,000)**	
**Circulatory system (I00-I99)**	351.34	301.63	400.99	582.55	1.93(1.88–1.98)
Cerebrovascular disease	193.86	165.27	260.32	369.75	2.24(2.16–2.31)
Ischemic heart disease	118.33	102.80	86.81	128.44	1.25(1.19–1.31)
**Neoplasms (C00-D48)**	257.23	220.77	240.01	286.61	1.30(1.26–1.34)
Lung	110.94	94.61	46.95	74.31	0.79(0.75–0.83)
Liver	39.26	34.34	49.02	53.44	1.56(1.44–1.68)
Stomach	26.98	22.81	45.16	56.22	2.47(2.25–2.70)
Colon and rectum	19.92	16.93	8.04	9.85	0.58(0.51–0.66)
Esophagus	22.54	19.69	13.80	23.00	1.17(1.05–1.30)
Breast	0.43	0.36	0.25	0.32	0.85(0.38–1.87)
**Respiratory system (J00-J99)**	82.28	70.15	134.23	215.28	3.07(2.92–3.23)
Chronic lower respiratory disease	61.49	52.09	130.52	210.83	4.05(3.82–4.29)
**Digestive system (K00-K93)**	26.76	23.25	21.03	24.62	1.06(0.96–1.17)
Diseases of liver	12.83	11.25	11.29	12.08	1.07(0.93–1.24)
**Endocrine, nutritional, and metabolic diseases (E00-E90)**	25.55	21.87	10.05	13.31	0.61(0.54–0.68)
Diabetes mellitus	24.44	20.91	9.64	12.80	0.61(0.55–0.69)

**Table 4:** Mortality rate of chronic disease among women in urban and rural areas in Hubei, 2008–2010

**Diseases**	**Urban**		**Rural**		**SRR (95% CI)**
	**Crude death rate**	**Standardized death rate**	**Crude death rate**	**Standardized death rate**	
	**(per 100,000)**	**(per 100,000)**	**(per 100,000)**	**(per 100,000)**	
**Circulatory system (I00-I99)**	315.50	212.08	324.99	392.38	1.85(1.79–1.91)
Cerebrovascular disease	180.40	122.36	202.00	242.90	1.99(1.91–2.07)
Ischemic heart disease	100.03	65.76	71.81	86.87	1.32(1.25–1.40)
**Neoplasms (C00-D48)**	148.97	113.98	113.74	125.14	1.10(1.05–1.15)
Lung	42.42	31.77	16.93	24.74	0.78(0.71–0.85)
Liver	12.33	9.25	16.95	18.27	1.98(1.71–2.29)
Stomach	11.68	9.04	19.38	21.79	2.41(2.08–2.79)
Colon and rectum	19.32	14.39	6.72	7.35	0.51(0.44–0.59)
Esophagus	7.34	5.38	4.99	8.05	1.50(1.23–1.82)
Breast	14.05	11.67	6.37	8.51	0.73(0.63–0.85)
**Respiratory system (J00-J99)**	45.62	30.48	77.72	97.06	3.18(2.95–3.44)
Chronic lower respiratory disease	31.21	20.85	75.64	94.68	4.54(4.14–4.98)
**Digestive system (K00-K93)**	18.55	13.08	7.71	8.61	0.66(0.57–0.76)
Diseases of liver	8.18	6.15	2.88	3.20	0.52(0.42–0.65)
**Endocrine, nutritional, and metabolic diseases (E00-E90)**	32.09	22.57	6.40	6.95	0.31(0.27–0.35)
Diabetes mellitus	30.45	21.47	6.80	7.34	0.34(0.30–0.39)

Almost 30% of deaths caused by NCDs occurred among citizens under the age of 60. Tables 5 and 6 show the YLL, AYLL and YLL rate among men and women in urban and rural areas. Circulatory system diseases and neoplasms accounted for most YLL rates in both men and women, and the AYLL caused by neoplasms was higher than AYLL caused by circulatory system diseases. The rate of life lost from breast cancer among women in urban areas was higher than among women in rural areas. Liver cancer accounted for the highest AYLL, while breast cancer had the highest AYLL among women. Although chronic lower respiratory disease mortality was higher in rural residents than in urban residents, the AYLL from this disease was lower in rural areas because it affected relatively older people.

**Table 5:** Years of life lost and mortality rate among men in urban and rural areas of Hubei, 2008–2010

**Diseases**	**Urban**			**Rural**		
	**Years of life**	**Average years of**	**Rate of life lost**	**Years of life lost**	**Average years of**	**Rate of life lost**
	**lost**	**life lost**			**life lost**	
**Circulatory system (I00-I99)**	52228	13.51	23.29	167745	12.32	28.23
Cerebrovascular disease	27228	12.49	12.14	107756	12.04	18.13
Ischemic heart disease	18481	14.38	8.24	37994	13.47	6.39
**Neoplasms (C00-D48)**	58902	14.56	26.26	171378	15.54	28.84
Lung	16560	12.84	7.38	43074	14.14	7.25
Liver	12558	17.68	5.60	45828	18.01	7.71
Stomach	6368	14.26	2.84	27452	13.75	4.62
Colon and rectum	3405	12.05	1.52	5754	16.18	0.97
Esophagus	2635	15.39	1.18	16186	11.99	2.72
Breast	30	14.83	0.01	225	15.00	0.04
**Respiratory system (J00-J99)**	4964	10.16	2.21	30171	8.78	5.08
Chronic lower respiratory disease	3296	9.28	1.47	28281	8.59	4.76
**Digestive system (K00-K93)**	7017	17.79	3.13	15151	15.10	2.55
Diseases of liver	4575	18.83	2.04	10340	17.18	1.74
**Endocrine, nutritional, and metabolic diseases (E00-E90)**	4712	14.79	2.10	6232	15.97	1.05
Diabetes mellitus	4362	14.40	1.95	5646	15.32	0.95

**Table 6:** Years of life lost and mortality rate among women in urban and rural areas of Hubei, 2008–2010

**Diseases**	**Urban**			**Rural**		
	**Years of life lost**	**Average years of**	**Rate of life lost**	**Years of life**	**Average years of**	**Rate of life lost**
		**life lost**		**lost**	**life lost**	
**Circulatory system (I00-I99)**	17306	8.71	7.93	96287	12.00	17.68
Cerebrovascular disease	10986	8.84	5.03	63001	12.22	11.57
Ischemic heart disease	3738	7.35	1.71	20648	12.36	3.79
**Neoplasms (C00-D48)**	31034	14.44	14.22	83619	18.00	15.35
Lung	4775	11.39	2.19	12839	16.59	2.36
Liver	2565	13.46	1.18	11793	16.35	2.17
Stomach	2303	13.95	1.06	10932	16.10	2.01
Colon and rectum	3241	12.89	1.49	4747	18.00	0.87
Esophagus	376	12.33	0.17	4220	10.66	0.77
Breast	5376	18.89	2.46	6960	22.61	1.28
**Respiratory system (J00-J99)**	3002	12.71	1.38	11693	8.86	2.15
Chronic lower respiratory disease	1916	11.78	0.88	10734	8.48	1.97
**Digestive system (K00-K93)**	1844	10.84	0.85	5067	19.74	0.93
Diseases of liver	1135	9.31	0.52	1893	16.56	0.35
**Endocrine, nutritional, and metabolic diseases (E00-E90)**	2817	8.94	1.29	4216	14.99	0.77
Diabetes mellitus	2711	8.82	1.24	4988	16.43	0.92

## Discussion

The present study identified that 86.2% of total deaths for people aged over 15 years were attributable to NCDs in Hubei during 2008–2010. This proportion is similar to the national average rate, and higher than in Brazil and Southeast Asia [20,21]. China has experienced an epidemiological transition from infectious to chronic disease, through a series of health interventions such as increased vaccination coverage, sanitation and water quality, better hygiene, improved access to medical care, and an advance in social and living standards [5]. The prevalence of risk factors for NCDs have increased, accompanying the rapid growth of the national economy and socio-environmental changes [22]. In addition, the decline of mortality and birth rates has increased the proportion of elderly people in China. It is notable that 6.3% of residents were aged 65 years or older in Hubei Province in 2000 [23], and the proportion increased to 9.1% in 2010 [16]. The ageing population likely contributed to an overall increase in chronic disease death rates.

We found that cerebrovascular diseases, ischemic heart disease and neoplasms were the main causes of death in both urban and rural areas. Overall, in both men and women, the standardized mortality rates were higher in rural than in urban areas. Previous studies have indicated that the prevalence rates of risk factors for chronic diseases such as cigarette and alcohol use are higher in rural than in urban areas in Hubei [13], but the chronic disease prevalence rates were higher in urban areas [17]. The higher death rates in rural areas may be a result of lower education and income levels, absence of public awareness and lack of resources for people to access health-related knowledge. In addition, health services in rural areas are poorer and residents often do not present at clinics for treatment of diseases, which can lead to higher mortality rates.

The standardized mortality rate of neoplasms among urban residents in Hubei was relative higher than in Xuzhou, while no significant differences were seen between rural residents of the two areas [24]. Among rural residents, the major cause of mortality from cancer was liver cancer, which may be due to a high consumption of salted, smoked, and chemically preserved foods. The highest discrepancy in mortality rates in urban areas between Hubei and Xuzhou was seen for lung cancer; mortality from lung cancer was also higher in urban areas than rural areas in Hubei Province. Environmental exposure in urban industrial areas has been identified as the major cause of lung cancer [25,26]. Similar to the rest of China, urban cities in Hubei Province have experienced severe air pollution problems in recent years. Many studies have shown that air pollution and other environmental factors can increase the risk of lung cancer [27]. The dramatic increase in air pollution in urban areas has been followed by a large increase in the incidence and death of lung cancer [28]. Further investigations on environmental exposure and genetic susceptibility need to be conducted to explain the high lung cancer mortality in Hubei.

Diabetes mellitus increases the risk of cardiovascular disease and premature death, and the prevalence of diabetes is high and increasing in China [29,30]. A higher prevalence of diabetes among urban residents than rural residents has been observed in developing countries throughout the world [31,32]. The standardized mortality from diabetes mellitus in Hubei is also significantly higher in urban areas than in rural areas. Urbanization, associated with changes in lifestyle that lead to physical inactivity, an unhealthy diet, and obesity have all been implicated as contributing factors in the development of diabetes.

Years of life lost is one of the methods for estimating the duration of time lost because of premature death and the burden of premature mortality. Years of life lost considers the number of deaths and also takes into account the age at which deaths occur. Calculating the average years of life lost involves estimating the average years a person would have lived if he or she had not died prematurely. Tumor of the breast and digestive organs, diseases of the liver and diabetes mellitus caused more years of life lost by premature death on individual level than other causes. However, the age distribution could influence the comparison of rates of life lost between rural and urban areas, which need to be explored in future studies.

Chronic diseases affect a much higher proportion of people during their prime working years in China, compared with developed countries [33]. The direct costs for these diseases include expenditures for admissions to the hospital, medical costs, nursing and family support. Indirect economic costs such as lower productivity, sick leave, and loss of productive workers caused by chronic diseases are already substantial and are likely to grow. In the next 10 years, NCDs are projected to cost China $558 billion [34]. Although the government has made extraordinary progress in reducing the number of people living in poverty in the last few decades, chronic NCDs are threatening this progress and exposing individuals, families and communities to stress.

Limitations of this study include the incompleteness of the DSPs data, which may underestimate the true mortality. Another problem is the misclassification of diseases. Although we trained the workers very carefully in data collection and used a strict quality control supervision strategy, it was not possible to assign a specific cause for some of deaths, or to avoid some misclassification like cardiovascular disease caused by diabetes mellitus. Finally, our study is limited by a lack of information about socioeconomic status, educational level, lifestyle risk factors, and occupational history, which prevented us from understanding the cause of urban–rural differences in mortality.

Nevertheless, our findings demonstrate that the residents in both urban and rural Hubei are at high risk for chronic diseases and these diseases impose a disproportionate burden on populations. Fortunately, these diseases can be substantially decreased by reducing the prevalence of their risk factors, early detection and timely treatments. Approaches include intervention of major risk factors for chronic diseases, primary, secondary and tertiary prevention, health promotion and related programs. An integrated approach across different sectors and disciplines should be taken to reduce morbidity and premature mortality of NCDs in Hubei.

## Conclusions

In this study, we showed that chronic diseases were the major cause of death and made up a large proportion of the disease burden in Hubei. Age standardized death rates due to NCDs were generally higher in rural than urban areas. Almost 30% of deaths caused by NCDs were in people under the age of 60, with potentially serious consequences for productivity and socioeconomic development. While circulatory system diseases were the leading causes of death in both urban and rural areas, our study highlights that attention should also be paid to breast cancer among women and chronic lower respiratory disease among rural residents, considering the high average years of potential life lost because of these diseases. The results reported here indicate the importance of governments focusing attention on this public health issue, and implementing comprehensive prevention strategies and sustained interventions to control these diseases.

## Abbreviations

NCDs: Chronic non-communicable diseases; DSPs: Disease surveillance points system; MOH-VR: Ministry of health-vital registration system; SRR: Standardized rate ratios; YLL: Years of potential life lost; AYLL: Average years of potential life lost.

## Competing interests

The authors declare that they have no competing interests.

## Authors’ contributions

QJZ and SFN designed the study. LWC, LT, LZ, SW, LL (Li Liu) oversaw data collection. Analysis was undertaken by LWC, LT, LL (Lu Long), JZ and YQW, under the supervision of SFN. LWC drafted the manuscript. All authors contributed to the interpretation of the data and approved the final manuscript. QJZ and SFN is the guarantor for the study. All authors read and approved the final manuscript.

## Pre-publication history

The pre-publication history for this paper can be accessed here:

http://www.biomedcentral.com/1471-2458/13/713/prepub
